# Comparison of HLA allelic imputation programs

**DOI:** 10.1371/journal.pone.0172444

**Published:** 2017-02-16

**Authors:** Jason H. Karnes, Christian M. Shaffer, Lisa Bastarache, Silvana Gaudieri, Andrew M. Glazer, Heidi E. Steiner, Jonathan D. Mosley, Simon Mallal, Joshua C. Denny, Elizabeth J. Phillips, Dan M. Roden

**Affiliations:** 1 Department of Pharmacy Practice and Science, University of Arizona College of Pharmacy, Tucson, Arizona, United States of America; 2 Division of Clinical Pharmacology, Department of Medicine, Vanderbilt University Medical Center, Nashville, Tennessee, United States of America; 3 Department of Biomedical Informatics, Vanderbilt University School of Medicine, Nashville, Tennessee, United States of America; 4 Division of Infectious Diseases, Department of Medicine, Vanderbilt University Medical Center, Nashville, Tennessee, United States of America; 5 School of Anatomy, Physiology and Human Biology, University of Western Australia, Nedlands, Western Australia, Australia; 6 Institute for Immunology & Infectious Diseases, Murdoch University, Murdoch, Western Australia, Australia; 7 Department of Pathology, Microbiology and Immunology, Vanderbilt University School of Medicine, Nashville, Tennessee, United States of America; 8 Department of Pharmacology, Vanderbilt University School of Medicine, Nashville, Tennessee, United States of America; University of Alabama at Birmingham, UNITED STATES

## Abstract

Imputation of human leukocyte antigen (HLA) alleles from SNP-level data is attractive due to importance of HLA alleles in human disease, widespread availability of genome-wide association study (GWAS) data, and expertise required for HLA sequencing. However, comprehensive evaluations of HLA imputations programs are limited. We compared HLA imputation results of HIBAG, SNP2HLA, and HLA*IMP:02 to sequenced HLA alleles in 3,265 samples from BioVU, a de-identified electronic health record database coupled to a DNA biorepository. We performed four-digit HLA sequencing for *HLA-A*, *-B*, *-C*, *-DRB1*, *-DPB1*, and *-DQB1* using long-read 454 FLX sequencing. All samples were genotyped using both the Illumina HumanExome BeadChip platform and a GWAS platform. Call rates and concordance rates were compared by platform, frequency of allele, and race/ethnicity. Overall concordance rates were similar between programs in European Americans (EA) (0.975 [SNP2HLA]; 0.939 [HLA*IMP:02]; 0.976 [HIBAG]). SNP2HLA provided a significant advantage in terms of call rate and the number of alleles imputed. Concordance rates were lower overall for African Americans (AAs). These observations were consistent when accuracy was compared across HLA loci. All imputation programs performed similarly for low frequency HLA alleles. Higher concordance rates were observed when HLA alleles were imputed from GWAS platforms versus the HumanExome BeadChip, suggesting that high genomic coverage is preferred as input for HLA allelic imputation. These findings provide guidance on the best use of HLA imputation methods and elucidate their limitations.

## Introduction

The major histocompatibility complex (MHC) and human leukocyte antigen (HLA) genes are extensively studied due to their key role in immune response.[1] Human Leukocyte Antigen (HLA) alleles have been implicated as risk factors for autoimmune diseases, infections, cancer, and immune-mediated adverse drug reactions. The HLA region is characterized by high linkage disequilibrium and a small number of single nucleotide polymorphisms (SNPs) can be used to tag the majority of HLA alleles.[2] Consequently, HLA alleles are frequently imputed from SNP-level data due to the widespread availability of genome-wide association study (GWAS) data and the expense and expertise required to directly sequence HLA loci with four digit resolution.[2] While HLA imputation programs have been applied successfully, notably in disease immunopathology,[3,4,5] comparisons of the performance of commonly used programs are limited.[6] In addition, the performance of HLA imputation programs with respect to the effect of genotyping platform, race/ethnicity, and frequency of HLA alleles is not well studied.

Multiple approaches that impute HLA alleles from single nucleotide polymorphism (SNP)-level data are available. Imputation methods include (1) HLA Genotype Imputation with Attribute Bagging (HIBAG), which employs multiple expectation-maximization-based classifiers to estimate the likelihood of HLA alleles;[7] (2) HLA*IMP:02 which uses a haplotype graph-based approach based on SNP data from multiple populations that can accommodate haplotypic diversity;[8] and (3) SNP2HLA which uses the imputation software package BEAGLE to impute both HLA alleles and the amino acid substitutions for those classical alleles.[9] These programs are freely available and have been used in published reports.[3,4,5]

Although the accuracy of these HLA imputation programs has been compared to sequence data, few previous studies have directly compared their relative accuracies.[6,10] These studies report varying results, were conducted in small homogeneous populations, looked only at class II alleles, and do not test the effect of SNP genotyping platform, race/ethnicity, and HLA allele frequency on imputation accuracy.[5,11,12] The present study compares imputation accuracy of three widely-used programs in a large population with both European and African ancestries. This comparison is necessary to guide the optimal application of these programs and elucidate their limitations.

## Materials and methods

### Study population

The study population was identified in BioVU, the Vanderbilt DNA databank that links DNA extracted from discarded blood samples to de-identified electronic health records (EMRs).[13] BioVU patients were enrolled from the Vanderbilt University Medical Center in Nashville, TN. The study population was selected from the Vanderbilt Electronic Systems for Pharmacogenomic Assessment (VESPA) cohort, which aims to analyze DNA samples from the BioVU database and EMRs to investigate the genetic underpinning for disease and drug response.[14,15] This study was approved by the Institutional Review Board at Vanderbilt University as described previously.[13,15]

### HLA typing

Sequence based typing on a deep sequencing platform is currently considered the gold standard for class I and II high resolution. HLA typing High resolution, four-digit HLA sequencing was performed for *HLA-A*, *HLA-B*, *HLA-C*, *HLA-DRB1*, *HLA-DPB1*, and *HLA-DQB1* at the Institute for Immunology and Infectious Diseases (IIID) at Murdoch University in Perth, Australia. The IIID is accredited by the American Society for Histocompatibility and Immunogenetics (ASHI) and the National Association of Testing Authorities (NATA) and the pipeline described below has been used in multiple previous studies.[16,17] Specific HLA Loci were PCR amplified using sample specific MID-tagged primers that amplify polymorphic exons from class I (A, B, C exons 2 and 3) and class II (DQ, exons 2 and 3; DRB and DPB1, exon 1) HLA loci. MID tagged primers have been optimized to minimize allele dropouts and primer bias. Amplified DNA products from unique MID tagged products (up to 48 MIDs) were pooled in equimolar ratios and subjected to library preparation, quantitation and emulsion PCR suitable for entry into the 454 FLX sequencing pipeline for long read sequencing. Clonally enriched beads were sequenced using 454 Titanium chemistry on a 454 FLX+ sequencer. Sequences were separated by MID tags and alleles called using an in house accredited HLA allele caller software pipeline that minimizes the influence of systematic sequencing errors in 454 data. Alleles were called using the latest IMGT HLA allele database as the allele reference library. Sample to report integrity were tracked and checked using proprietary and accredited Laboratory Information and Management System (LIMS) and HLA analysis reporting software that performs comprehensive allele balance and contamination checks on the final dataset. All samples that were successfully typed were included in the study population.

### SNP-level genotyping

All samples included in this study population (n = 3,265) were genotyped using a genome-wide platform, either the Illumina^®^ HumanOmni1-QUAD (n = 2,430 [74%]) or HumanOmni5-QUAD BeadChip (n = 835 [26%]). The HumanOmni1-QUAD contains 11,675 SNPs in the HLA region and the HumanOmni5-QUAD contains 26,952 SNPs in the HLA region (GRCh37 chr6:28,477,797–33,448,354). In addition, 96% of the samples (n = 3,152) were also typed using the Illumina^®^ HumanExome BeadChip, which contains putative functional exonic variants and a small amount of non-exonic content including 2,061 HLA tagging SNPs. SNP data from both the HumanExome BeadChip and GWAS platforms were cleaned using the quality control (QC) pipeline developed by the eMERGE Genomics Working Group.[18,19] Samples were classified as being of European or African descent (≥90% European ancestry for European decent and ≥80% African ancestry for African decent) using ancestry informative markers (AIMs) from genome-wide platforms input into STRUCTURE using Hapmap reference populations.[20] To further assess admixture, principal components analysis (PCA) was also performed on GWAS data and compared to PCA generated using 1000 Genomes samples.

### HLA allele imputation

Classical four digit HLA alleles were imputed from SNP data from HumanExome BeadChip and GWAS platforms using HIBAG version 3,[7] HLA*IMP:02,[8] and SNP2HLA (8/7/2102).[9] The Type 1 Diabetes Genetics Consortium (T1DGC) reference panel was used for SNP2HLA and HIBAG whereas HLA*IMP:02 uses an internal reference panel. Individual dosages for classical 4-digit alleles at *HLA-A*, *-B*, *-C*, *-DQA1*, *-DQB1* and *-DRB1* were imputed. A posterior probability (PP) cutoff of 0.5 was implemented for imputed alleles based on previous literature.[6,21] The three HLA imputation software programs were compared to sequenced HLA alleles using the latest available version of each program. A sensitivity analysis was also performed to account for imputed alleles with similar PPs. In our primary analysis, a given sample could be assigned one imputed allele with a PP of 0.51 and another imputed allele with a PP of 0.49, indicating minimal confidence of one imputed allele over the other. To exclude such imputations, the highest and second highest PP was used to calculate a posterior probability ratio and HLA allele calls were excluded if this ratio was less than 1.5.

### Statistical analysis

The primary assessment metrics for each imputation program were concordance with sequenced HLA type results and call rate. Call rate was defined as the number of imputed alleles divided by the total number of individuals for which imputation was attempted. Concordance was defined as the number of imputed four digit alleles matching sequenced alleles divided by the total number of imputed alleles within the population. The calculation of concordance thereby did not consider individuals for which alleles were not imputed. For instance, if an imputation program did not impute an allele for an individual, this would not affect the concordance rate, but the call rate would be decreased for that imputation program. The total number of HLA alleles imputed by each program is also reported, which did not require an individual within the population to possess that allele. We assessed the relative accuracy of the three imputation programs and the robustness of each program to differences in race/ethnic group (European versus African ancestry), SNP genotyping platform (HumanExome BeadChip, HumanOmni1-QUAD, and HumanOmni5-QUAD), and frequency of HLA allele (minor allele frequency less than 0.05 and 0.01).

## Results

**Fig 1:**
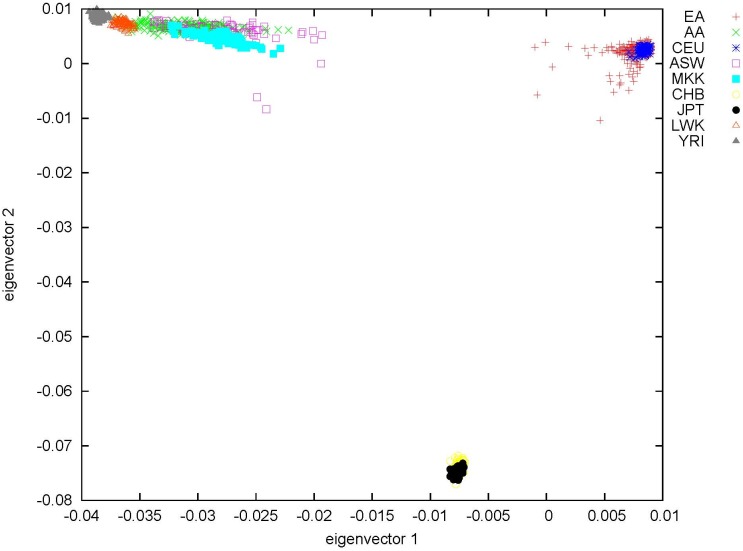
Principal components analysis of 1000 Genomes samples and study population. Eigenvectors 1 and 2 are plotted to determine racial decent and admixture of European and African Americans in the BioVU population. EA indicates European American (BioVU); AA, African American (BioVU); CEPH, 1000 Genomes Utah Residents; ASW, Americans of African Ancestry in Southwestern USA; MKK, Maasai in Kinyawa, Kenya; CHB, Han Chinese in Bejing, China; JPT, Japanese in Tokyo, Japan; LWK, Luhya in Webuye, Kenya; YRI, Yoruba in Ibadan, Nigeria.

Our study population (n = 3,265) included 1,592 females (48.8%) and had an average age of 57.8 (standard deviation 20.8) years. Our population was comprised of 2,947 European Americans (EAs) (90.2%) and 318 African Americans (AAs) by Structure-defined race. The average percent European, African, and Asian ancestry for the European ancestry study population was 98.2%, 1.0%, and 0.8% respectively and for the African ancestry study population, these percentages were 17.7%, 79.1%, and 3.3%, respectively. Principal components analysis suggested limited admixture in both the European and African ancestry study populations. (Fig 1)

SNP2HLA provided imputations for the largest overall number of HLA alleles at 210 compared to HLA*IMP:02 (140 alleles) and HIBAG (175 alleles). (Table 1) The performance was excellent in EAs with overall concordance greater than 93% for all three programs. However, performance was poorer in AAs with reduced concordance rates (0.919 [SNP2HLA]; 0.619 [HLA*IMP:02]; 0.929 [HIBAG]). The overall concordance rate compared to HLA sequencing was highest for HIBAG (97.6% in EAs and 92.9% in AAs) and SNP2HLA (97.5% in EAs and 91.9% in AAs) compared to HLA*IMP:02 (93.9% in EAs and 61.9% in AAs). The overall call rate was highest for SNP2HLA. (Table 1)

**Table 1:** HLA imputation programs evaluation for all HLA alleles.

Race/Ethnicity	Imputation Program	Concordance Rate	Call Rate	Predicted Alleles (n)
European Americans (n = 2,947)^1^	SNP2HLA	0.975	1.00	210
	HLA*IMP:02	0.939	0.985	140
	HIBAG	0.976	0.978	175
African Americans (n = 318)^2^	SNP2HLA	0.919	0.999	174
	HLA*IMP:02	0.619	0.768	134
	HIBAG	0.929	0.584	131

Concordance and call rates generated from imputed alleles with posterior probability>0.50 versus sequenced alleles after combining data for HumanOmni1-QUAD and HumanOmni5-QUAD platforms by race/ethnicity.

^1)^ Based on sequencing, 325 distinct four digit alleles were present in the European American population.

^2)^ Based on sequencing, 219 distinct four digit alleles were present in the African American population.

The concordance rate by HLA loci imputed from GWAS platforms (HumanOmni1-QUAD and HumanOmni5-QUAD) ranged from 98.8% for SNP2HLA and HIBAG in *HLA-DQB1* in EAs and 41.4% for HLA*IMP:02 in *HLA-DRB1* in AAs. (Table 2) All programs had higher concordance rates and call rates in EAs compared to AAs, although consistently higher call rates were observed with SNP2HLA and consistently lower call and concordance rates were observed with HLA*IMP:02. Class I and class II HLA alleles were imputed with similar accuracy by all programs in EAs. In the sensitivity analysis implementing a posterior probability ratio cutoff of 1.5, we observed slightly increased concordance rates and slightly decreased call rates for each imputation program overall and by HLA locus. (S1 File)

**Table 2:** Concordance rate and call rate for each imputation program.

		European Americans		African Americans	
Allele	Imputation Program	Concordance Rate	Call Rate	Concordance Rate	Call Rate
*HLA-A*	SNP2HLA	0.983	0.999	0.969	0.995
	HLA*IMP:02	0.963	0.997	0.675	0.855
	HIBAG	0.986	0.996	0.960	0.796
*HLA-B*	SNP2HLA	0.969	1.00	0.884	1.00
	HLA*IMP:02	0.952	0.979	0.423	0.752
	HIBAG	0.978	0.967	0.953	0.403
*HLA-C*	SNP2HLA	0.987	1.00	0.884	1.00
	HLA*IMP:02	0.984	0.994	0.792	0.741
	HIBAG	0.987	0.992	0.957	0.619
*HLA-DPB1*	SNP2HLA	0.957	1.00	0.945	1.00
	HLA*IMP:02	0.829	0.987	0.567	0.708
	HIBAG	0.957	0.975	0.834	0.475
*HLA-DQB1*	SNP2HLA	0.988	1.00	0.907	1.00
	HLA*IMP:02	0.983	0.993	0.845	0.761
	HIBAG	0.988	0.990	0.904	0.654
*HLA-DRB1*	SNP2HLA	0.964	1.00	0.920	1.00
	HLA*IMP:02	0.924	0.961	0.414	0.791
	HIBAG	0.959	0.946	0.946	0.557

Concordance and call rates generated from imputed alleles with posterior probability>0.50 versus sequenced alleles after combining data for HumanOmni1-QUAD and HumanOmni5-QUAD platforms by HLA locus and race/ethnicity.

When divided by platform, HLA imputation for each program had the highest concordance when the HumanOmni5-QUAD platform, which included the largest number of genotyped SNPs and the most comprehensive coverage in the HLA region, was used as input versus HumanOmni1-QUAD. (Table 3) Concordance rates were lower when HumanExome BeadChip data was used as input. SNP2HLA and HIBAG maintained high concordance rates despite the loss of genomic coverage associated with the HumanExome BeadChip, whereas HLA*IMP:02 showed a greater decrease in concordance. These observations were consistent in both EAs and AAs. All imputation programs performed well for low frequency alleles with little differences in concordance rate for frequency<0.05 (0.981 [SNP2HLA]; 0.951 [HLA*IMP:02]; 0.975 [HIBAG]) and for frequency<0.01 (0.979 [SNP2HLA]; 0.945 [HLA*IMP:02]; 0.971 [HIBAG]). (Table 3)

**Table 3:** Concordance rates and call rates for imputation programs for all HLA loci by platform and allele frequency in European Americans.

		SNP2HLA	HLA*IMP:02	HIBAG
Platform	HumanExome BeadChip	.969/.999	.892/.950	.976/.973
	HumanOmni1-QUAD	.975/1.00	.939/.985	.976/.978
	HumanOmni5-QUAD	.975/1.00	.938/.985	.975/.977
	HumanOmni1-QUAD / HumanOmni5-QUAD	.976/1.00	.942/.986	.979/.979
HLA allele Frequency^1^	Freq.<0.05	.981/-	.951/-	.975/-
	Freq.<0.01	.979/-	.945/-	.971/-

Freq. indicates frequency cutoff; HLA, human leukocyte antigen.

^1)^ Call rates not estimated when frequency cutoffs were implemented

**Fig 2:**
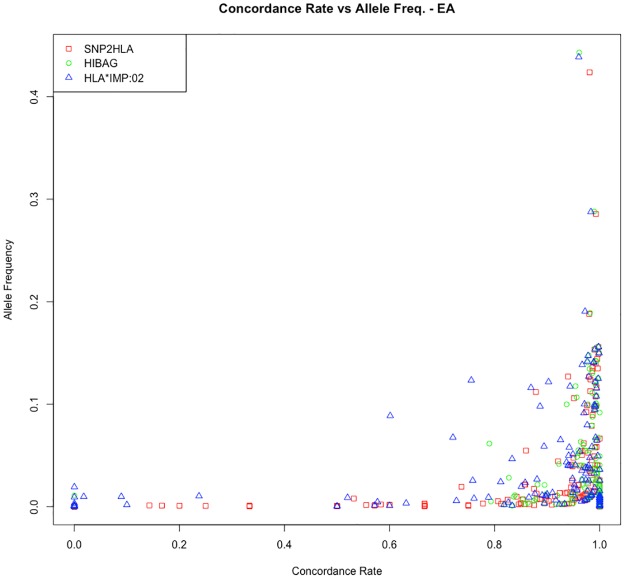
Allele frequency versus concordance rates of HLA alleles by imputation program in European Americans. Concordance rates were generated using OMNI1 and OMNI5 combined SNP-level data and posterior probability >0.50 for each imputation program.

**Fig 3:**
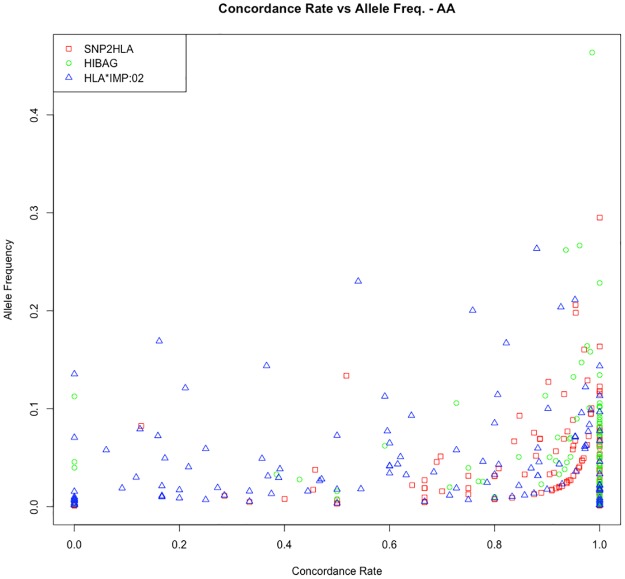
Allele frequency versus concordance rates of HLA alleles by imputation program in African Americans. Concordance rates were generated using OMNI1 and OMNI5 combined SNP-level data and posterior probability>0.50 for each imputation program.

Figs 2 and 3 show the frequency of imputed plotted against concordance rates by imputation program in EAs and AAs. These figures suggest that HLA*IMP:02 underperformed in terms of accuracy versus the SNP2HLA and HIBAG in both EAs and AAs. Although the majority of low frequency alleles had high concordance rates, alleles with poor concordance to sequence data were likely to be low frequency alleles. Concordance rates for individual alleles are listed in the Supplemental Materials (S1 File). Table 4 compares concordance and call rates of common HLA alleles previously associated with autoimmune disease and adverse drug reactions. These disease-associated alleles had high concordance rates for all imputation programs in EAs, but a large decrease in accuracy was observed for disease-associated allele in AAs.

**Table 4:** Concordance rate and call rate for important disease-associated and adverse drug reaction-associated alleles.

HLA Allele	Disease/ADR	Imputation Program	Concordance Rate (EAs)^1^	Concordance Rate (AAs)^1^
B*27:*05*	ankylosing spondylitis[24]	SNP2HLA	0.948	0.667
		HLA*IMP:02	0.936	0.250
		HIBAG	0.933	1.000
B*57:*01*	abacavir HSN[17]; flucloxacillin DILI[25]	SNP2HLA	0.996	1.000
		HLA*IMP:02	0.978	0.118
		HIBAG	0.975	1.000
B*58:*01*	allopurinol SJS/TEN[26]	SNP2HLA	1.000	0.857
		HLA*IMP:02	1.000	0.621
		HIBAG	0.964	0.783
DQB1*02:*01*	Sjogren’s Syndrome[27]	SNP2HLA	0.980	0.518
		HLA*IMP:02	0.997	0.957
		HIBAG	0.997	0.698
DRB1*03:*02*	Lupus erythematosus[28]	SNP2HLA	0.750	1.000
		HLA*IMP:02	-	-
		HIBAG	1.000	0.951
DRB1*08:*01*	primary biliary cirrhosis[29]	SNP2HLA	0.978	1.000
		HLA*IMP:02	0.882	0.154
		HIBAG	0.951	-
DRB1*04:*01*	rheumatoid arthritis[30]	SNP2HLA	0.951	0.889
		HLA*IMP:02	0.856	0.179
		HIBAG	0.927	0.158

Concordance rates were generated using HumanOmni1-QUAD and HumanOmni5-QUAD combined SNP-level data and posterior probability>0.50 for each imputation program by HLA locus and race/ethnicity. HLA indicates human leukocyte antigen; EA, European American; AA, African American; HSN, hypersensitivity; DILI, drug-induced liver injury; NA, not applicable; SJS, Stevens-Johnson Syndrome; TEN, toxic epidermal necrosis

^1)^ “-”indicates that the imputation program did not impute the allele.

## Discussion

We provide a detailed evaluation and comparison of three commonly used HLA imputation programs. Overall, the programs performed similarly in terms of concordance with sequence data in EAs. We observed that HLA imputation accuracy was decreased in AAs and when using genotyping platforms with lower HLA coverage as input. However, SNP2HLA was observed to predict a greater number of HLA alleles with a higher call rate and was most robust when using a platform with limited genomic coverage and when imputing alleles in AAs.

Overall, we observed similar concordance rates to sequence results when compared with previous studies.[6,10,21] Our data are also consistent with previous studies which have shown that imputation accuracy was decreased in non-Caucasian populations.[6,22] The decrease in accuracy in AAs may have been due to a reduced linkage disequilibrium structure in this race group. Reduced imputation accuracy in AAs may also have been due to the use of the T1DGC as a reference panel, since the T1DGC consists primarily of patients of European descent and previous studies have shown that HLA imputation accuracy is highly dependent on the racial similarity between the test and reference populations.[6,10,21] If individuals in our population carry an HLA allele that is rare or absent from the individuals in the reference panel, the allele would not be imputed. Comparisons of HLA imputation programs in admixed populations are limited. For SNP2HLA, overall imputation accuracies for AAs in this study were high relative to other studies, possibly indicating high European admixture in the AA BioVU population. This observation suggests that SNP2HLA is preferred when an admixed population without a representative reference population is available.

We observed a higher HLA imputation accuracy for each program studied when input genotype data had greater coverage in the HLA region. Our data suggest a preference for genome-wide platforms with greater genomic coverage when imputing HLA alleles. However, HLA imputation was still high when data from the HumanExome BeadChip, which has 2,061 HLA tags, was used as input, suggesting that valuable information can still be gained in the absence of high coverage genome-wide platforms. Our results are consistent with a previous report for *HLA-DRB1* alleles in a Finnish population (n = 161) using HLA*IMP and SNP2HLA, which reported that SNP coverage and quality did not markedly affect HLA imputation results.[10] We found a slight but consistent increase in the accuracy of HLA imputation for all programs when genomic coverage of the input platform was increased. We also observed that SNP2HLA was the most robust program with respect to maintaining accuracy despite a loss of genomic coverage.

Although each of the three HLA imputation programs evaluated performed similarly, SNPHLA was observed to have the best accuracy call rates overall for most of the analyses performed. SNP2HLA provided a significant advantage in the number of alleles imputed and outperformed the other two programs in our AA population. The high number of alleles imputed with high accuracy offers advantages especially when uncommon alleles are included in analyses. SNP2HLA was also observed to maintain imputation accuracy when genomic coverage was decreased and when imputing alleles for AA individuals. These data suggest that SNP2HLA should be used in preference to HLA*IMP:02 and HIBAG in similar populations genotyped on similar platforms.

Since all programs tested had high concordance rates for most alleles in EAs, with the HumanExome BeadChip as input, and in low frequency alleles, selection of HLA programs based on other factors would be reasonable when HLA alleles are being imputed for a homogeneous EA population. Such factors might include data output, computing time, and availability and flexibility of appropriate reference panels. Although all programs impute all the alleles tested in this study, other alleles such as those in *HLA-DPA1* are not imputed in all programs. SNP2HLA also offers output which includes two digit HLA alleles, HLA amino acid changing polymorphisms, and phased output files. However, SNP2HLA has a computational restriction which may force sub-setting of data. In any case, imputation from SNP data may be useable in research setting with large numbers of samples but not likely to have accuracy to justify use in clinical practice.[23]

The strengths of our study include a large sample size relative to previous publications as well as the inclusion of multiple platforms with varying HLA region coverage. Unlike previous studies, we have performed an extensive set of comparisons within a single population, including both class I and II HLA alleles and multiple race groups, including an admixed US population. Our study has several limitations worthy of mention. A reference population specific to our AA population was not used and this likely contributed to reduced imputation accuracy. However, SNP2HLA was robust in terms of concordance rates in AAs, suggesting that the T1DGC reference population may be sufficient in an admixed US population of AAs. Our approach underscores the limited availability of appropriate reference panels of HLA alleles for non-Caucasian individuals. We also did not sequence *HLA-DQA1* and–*DPA1* alleles and, thus, no comparison was possible for these alleles, although the variability within these genes is known to be low. We also did not examine differences in strand concordance for HLA alleles among the HLA imputation programs. Sequence based typing on a deep sequencing platform is currently considered the gold standard for class I and II high resolution HLA typing, but it can be limited by the smaller number of laboratories that have this expertise and the expense and turnaround time of typing. Since only successfully sequenced samples were included, samples that might have been difficult to sequence were not reflected in our analysis. Although we did compare imputation accuracy using the HumanOmni5-QUAD and HumanOmni1-QUAD platforms as input, we did not genotype samples on both platforms and so these platforms were compared in different subsets of patients. Finally, the racial makeup of the BioVU population precluded an evaluation and comparison of HLA imputation methods in other race/ethnic groups such as Asians or Hispanics.

In most scenarios tested, SNP imputation programs performed similarly in terms of concordance. However, SNP2HLA typically had the highest concordance with robust call rates and provided a significant advantage in the number of alleles imputed. All programs resulted in better concordance in EAs versus AAs and performed similarly for low frequency alleles. Our results suggest that high genomic coverage is preferred as input for HLA allelic imputation. These observations are useful to provide guidance on the best use of HLA imputation methods and elucidate their limitations.

## Supporting information

S1 File Supplemental materials. (PDF)
