# Supplementary material for: Comparison of HLA allelic imputation programs
Source: PLoS One. 2017 Feb 16;12(2):e0172444. doi: 10.1371/journal.pone.0172444 (PMC5312875; doi:10.1371/journal.pone.0172444)
Supplement: S1 File — (PDF) [file pone.0172444.s001.pdf]

SUPPLEMENTAL MATERIALS

Comparison of HLA Allelic Imputation Programs

Jason H Karnes[1,2], Christian M Shaffer[2], Lisa Bastarache[3], Silvana Gaudieri[4,5,6], Andrew M Glazer[2], Heidi E Steiner[1], Jonathan D Mosley[2], Simon Mallal[7], Joshua C Denny[3], Elizabeth J Phillips[2,4,6], Dan M Roden[2,3,8]*

1) Department of Pharmacy Practice and Science, University of Arizona College of Pharmacy, Tucson, AZ
2) Division of Clinical Pharmacology, Department of Medicine, Vanderbilt University Medical Center, Nashville, TN
3) Department of Biomedical Informatics, Vanderbilt University School of Medicine, Nashville, TN
4) Division of Infectious Diseases, Department of Medicine, Vanderbilt University Medical Center, Nashville, TN
5) School of Anatomy, Physiology and Human Biology, University of Western Australia, Nedlands, Western Australia, Australia
6) Institute for Immunology & Infectious Diseases, Murdoch University, Murdoch, Western Australia, Australia
7) Department of Pathology, Microbiology and Immunology, Vanderbilt University School of Medicine, Nashville, TN
8) Department of Pharmacology, Vanderbilt University School of Medicine, Nashville, TN

*__Corresponding Author:__

Email: dan.roden@vanderbilt.edu

Table S1: HLA imputation programs evaluation for all HLA alleles and posterior probability ratio greater than 1.5

Race/Ethnicity	Imputation Program	Concordance Rate	Call Rate	Predicted Alleles (n)
European Americans (n=2,947)[1]	SNP2HLA<br>HLA*IMP:02<br>HIBAG	0.976<br>0.945<br>0.981	0.995<br>0.970<br>0.954	210<br>140<br>175
African Americans (n=318)[2]	SNP2HLA<br>HLA*IMP:02<br>HIBAG	0.912<br>0.681<br>0.954	1.00<br>0.887<br>0.471	174<br>134<br>131

Concordance and call rates generated from imputed alleles versus sequenced alleles after combining data for HumanOmni1-QUAD and HumanOmni5-QUAD platforms by race/ethnicity. HLA allele calls are restricted to posterior probability > 0.5 and posterior probability ratio>1.5. Posterior probability ratio was calculated as the ratio of highest and second highest posterior probability for an individual sample.

1) Based on sequencing, 325 distinct four digit alleles were present in the European American population.
2) Based on sequencing, 219 distinct four digit alleles were present in the African American population.

Table S2: Concordance rate and call rate for each imputation program by HLA locus and posterior probability ratio greater than 1.5

		European Americans		African Americans	
Allele	Imputation Program	Concordance Rate	Call Rate	Concordance Rate	Call Rate
HLA-A	SNP2HLA	0.984	0.997	0.978	0.991
	HLA*IMP:02	0.964	0.991	0.547	0.862
	HIBAG	0.986	0.989	0.970	0.726
HLA-B	SNP2HLA	0.973	0.989	0.886	0.989
	HLA*IMP:02	0.955	0.966	0.547	0.862
	HIBAG	0.982	0.943	0.969	0.305
HLA-C	SNP2HLA	0.987	0.998	0.842	1.00
	HLA*IMP:02	0.984	0.994	0.807	0.931
	HIBAG	0.988	0.987	0.979	0.516
HLA-DPB1	SNP2HLA	0.961	0.989	0.944	1.00
	HLA*IMP:02	0.844	0.942	0.607	0.772
	HIBAG	0.962	0.951	0.867	0.343
HLA-DQB1	SNP2HLA	0.989	0.999	0.907	0.995
	HLA*IMP:02	0.984	0.992	0.866	0.918
	HIBAG	0.991	0.977	0.947	0.472
HLA-DRB1	SNP2HLA	0.959	0.997	0.919	0.994
	HLA*IMP:02	0.932	0.936	0.515	0.892
	HIBAG	0.972	0.875	0.966	0.462

Concordance and call rates generated from imputed alleles versus sequenced alleles after combining data for HumanOmni1-QUAD and HumanOmni5-QUAD platforms by HLA locus and race/ethnicity. HLA allele calls are restricted to posterior probability > 0.5 and posterior probability ratio>1.5. Posterior probability ratio was calculated as the ratio of highest and second highest posterior probability for an individual sample.

Table S3: Frequencies and concordance rates for HLA alleles by imputation program and race/ethnicity[1,2]

	European Americans				African Americans			
**HLA allele**	**FREQ**	**SNP2HLA**	**HIBAG**	**HLA*IMP**	**FREQ**	**SNP2HLA**	**HIBAG**	**HLA*IMP**
**A*01:01**	0.156	0.998	0.998	0.998	0.039	0.960	0.750	0.800
**A*01:02**	0.000	NA	NA	NA	0.008	0.800	NA	NA
**A*01:03**	0.000	1.000	NA	NA	0.000	NA	NA	NA
**A*02:01**	0.286	0.993	0.990	0.983	0.095	0.983	0.980	0.983
**A*02:02**	0.001	0.750	1.000	NA	0.047	0.967	1.000	NA
**A*02:03**	0.001	0.000	NA	NA	0.000	NA	NA	NA
**A*02:05**	0.008	1.000	1.000	1.000	0.013	1.000	0.800	0.250
**A*02:06**	0.003	0.850	0.929	0.000	0.000	NA	NA	NA
**A*02:07**	0.000	0.000	NA	NA	0.000	NA	NA	NA
**A*02:11**	0.000	0.000	NA	NA	0.000	NA	NA	NA
**A*03:01**	0.144	0.995	0.979	0.978	0.095	0.983	1.000	0.902
**A*03:02**	0.002	0.929	NA	NA	0.000	NA	NA	NA
**A*11:01**	0.065	0.995	0.992	0.995	0.025	0.938	1.000	0.929
**A*23:01**	0.018	1.000	1.000	0.991	0.115	1.000	1.000	1.000
**A*24:02**	0.079	0.985	0.985	0.976	0.011	1.000	1.000	0.368
**A*24:03**	0.000	NA	NA	0.000	0.000	NA	NA	NA
**A*24:07**	0.000	0.000	NA	NA	0.000	NA	NA	NA
**A*24:10**	0.001	0.000	NA	NA	0.000	NA	NA	NA
**A*25:01**	0.021	0.857	0.882	0.758	0.003	0.500	NA	0.167
**A*26:01**	0.022	0.858	0.895	0.851	0.013	0.875	0.429	0.545
**A*29:01**	0.003	1.000	1.000	NA	0.000	NA	NA	NA
**A*29:02**	0.041	0.996	1.000	0.937	0.036	1.000	1.000	0.955
**A*30:01**	0.013	1.000	1.000	0.988	0.081	1.000	1.000	0.980
**A*30:02**	0.011	1.000	1.000	1.000	0.068	1.000	1.000	1.000
**A*30:04**	0.001	1.000	1.000	1.000	0.000	NA	NA	1.000
**A*31:01**	0.033	0.984	0.995	0.990	0.019	0.667	1.000	0.389
**A*32:01**	0.032	0.995	0.995	0.974	0.009	1.000	1.000	0.159
**A*33:00**	0.000	NA	NA	0.000	0.000	NA	NA	0.000
**A*33:01**	0.007	0.854	0.854	NA	0.019	1.000	1.000	NA
**A*33:03**	0.003	0.933	1.000	NA	0.066	1.000	1.000	NA
**A*34:02**	0.001	1.000	1.000	0.571	0.032	1.000	1.000	1.000
**A*36:01**	0.000	NA	NA	NA	0.016	1.000	0.769	NA
**A*43:86**	0.000	NA	NA	0.000	0.000	NA	NA	NA
**A*66:01**	0.003	1.000	1.000	NA	0.011	1.000	NA	NA
**A*66:02**	0.000	NA	NA	NA	0.000	NA	1.000	NA
**A*68:01**	0.037	0.991	0.995	0.973	0.044	1.000	1.000	0.596

Table S3 (continued)

	European Americans				African Americans			
**Allele**	**FREQ**	**SNP2HLA**	**HIBAG**	**HLA*IMP**	**FREQ**	**SNP2HLA**	**HIBAG**	**HLA*IMP**
**A*68:02**	0.010	0.983	1.000	0.891	0.062	1.000	1.000	0.972
**A*68:03**	0.000	0.000	NA	NA	0.000	NA	NA	NA
**A*69:01**	0.001	1.000	1.000	NA	0.000	NA	NA	NA
**A*74:01**	0.001	0.200	NA	NA	0.070	0.886	0.943	NA
**A*74:03**	0.000	NA	1.000	NA	0.000	NA	NA	NA
**A*80:01**	0.000	NA	NA	NA	0.000	1.000	1.000	NA
**B*07:02**	0.140	0.993	0.994	0.989	0.077	0.939	0.897	0.882
**B*07:05**	0.002	0.846	0.917	0.818	0.022	0.643	NA	0.273
**B*08:01**	0.116	0.994	0.993	0.994	0.047	0.967	1.000	0.885
**B*13:01**	0.000	NA	NA	NA	0.002	0.000	NA	NA
**B*13:02**	0.025	1.000	0.993	0.966	0.008	1.000	NA	0.444
**B*14:01**	0.011	0.984	1.000	0.984	0.006	1.000	NA	0.500
**B*14:02**	0.025	1.000	1.000	0.979	0.019	1.000	1.000	1.000
**B*15:01**	0.062	0.970	0.964	0.926	0.013	0.750	NA	0.217
**B*15:03**	0.003	1.000	1.000	0.833	0.064	1.000	1.000	1.000
**B*15:10**	0.001	1.000	1.000	NA	0.025	0.938	1.000	0.500
**B*15:16**	0.001	1.000	1.000	0.500	0.019	1.000	1.000	0.667
**B*15:17**	0.003	0.941	0.938	0.577	0.006	1.000	NA	0.800
**B*15:18**	0.003	0.938	0.929	0.933	0.002	1.000	NA	1.000
**B*15:25**	0.000	0.000	NA	NA	0.005	0.000	NA	NA
**B*18:01**	0.041	0.946	0.953	0.943	0.027	0.941	1.000	0.786
**B*18:03**	0.000	NA	NA	0.000	0.000	NA	NA	0.000
**B*27:02**	0.002	0.600	1.000	NA	0.000	NA	NA	0.000
**B*27:05**	0.039	0.948	0.965	0.940	0.009	0.667	NA	0.333
**B*27:07**	0.000	0.500	NA	NA	0.000	NA	NA	NA
**B*35:01**	0.054	0.966	0.974	0.894	0.069	0.886	0.944	0.366
**B*35:02**	0.007	0.854	0.878	0.917	0.000	NA	NA	NA
**B*35:03**	0.013	0.924	0.955	0.877	0.003	0.500	NA	0.200
**B*35:08**	0.003	0.667	0.864	NA	0.000	NA	NA	0.000
**B*35:12**	0.000	0.000	NA	NA	0.000	NA	NA	NA
**B*35:17**	0.000	NA	NA	NA	0.000	NA	NA	0.000
**B*37:01**	0.013	1.000	1.000	0.949	0.006	1.000	NA	0.750
**B*38:01**	0.012	0.944	0.983	0.900	0.000	NA	NA	0.000
**B*38:02**	0.001	0.500	NA	NA	0.000	NA	NA	NA
**B*39:01**	0.010	0.842	0.836	0.788	0.011	0.286	NA	0.167
**B*39:06**	0.005	0.903	0.950	0.727	0.002	1.000	1.000	0.000

Table S3 (continued)

	European Americans				African Americans			
**Allele**	**FREQ**	**SNP2HLA**	**HIBAG**	**HLA*IMP**	**FREQ**	**SNP2HLA**	**HIBAG**	**HLA*IMP**
**B*39:09**	0.000	NA	NA	NA	0.002	0.000	NA	NA
**B*39:10**	0.000	0.000	NA	0.000	0.006	1.000	1.000	NA
**B*39:24**	0.000	NA	1.000	0.000	0.000	NA	NA	0.000
**B*40:01**	0.067	1.000	0.997	0.992	0.009	1.000	NA	1.000
**B*40:02**	0.013	0.935	0.947	0.910	0.002	1.000	NA	1.000
**B*40:06**	0.001	0.167	1.000	NA	0.000	NA	NA	NA
**B*41:01**	0.002	1.000	1.000	0.632	0.008	0.400	NA	0.061
**B*41:02**	0.003	0.895	0.895	0.895	0.000	NA	NA	0.250
**B*42:01**	0.001	1.000	NA	NA	0.038	1.000	1.000	NA
**B*42:02**	0.000	NA	NA	NA	0.017	0.455	1.000	NA
**B*44:02**	0.098	0.993	0.993	0.990	0.016	0.700	NA	0.467
**B*44:03**	0.050	0.966	0.960	0.949	0.067	0.953	0.846	0.642
**B*44:04**	0.001	0.333	NA	0.000	0.002	0.000	NA	NA
**B*44:05**	0.002	0.857	0.857	0.923	0.000	NA	NA	0.000
**B*45:01**	0.008	0.960	0.979	0.955	0.036	0.957	1.000	0.900
**B*46:01**	0.000	NA	NA	NA	0.002	0.000	NA	NA
**B*47:01**	0.002	1.000	1.000	1.000	0.002	1.000	NA	1.000
**B*48:01**	0.000	0.500	NA	NA	0.000	NA	NA	NA
**B*49:01**	0.015	0.989	0.977	0.977	0.041	0.962	1.000	0.727
**B*50:01**	0.010	0.915	0.946	0.898	0.003	0.500	NA	0.333
**B*51:01**	0.048	0.950	0.957	0.941	0.038	0.458	0.500	0.357
**B*51:05**	0.000	NA	NA	NA	0.003	0.000	NA	NA
**B*51:08**	0.001	0.250	1.000	NA	0.002	0.000	NA	0.000
**B*52:01**	0.007	0.952	1.000	0.976	0.005	0.333	0.500	NA
**B*53:01**	0.005	1.000	1.000	1.000	0.118	1.000	0.976	0.971
**B*54:01**	0.000	1.000	NA	NA	0.000	NA	NA	NA
**B*55:01**	0.019	0.945	0.971	0.951	0.005	0.667	NA	1.000
**B*56:01**	0.007	0.881	0.947	0.761	0.003	1.000	NA	1.000
**B*56:03**	0.000	NA	NA	NA	0.002	0.000	NA	NA
**B*56:04**	0.000	NA	NA	NA	0.002	0.000	NA	NA
**B*57:01**	0.040	0.996	0.991	0.978	0.003	1.000	NA	0.118
**B*57:02**	0.000	NA	NA	NA	0.002	1.000	NA	NA
**B*57:03**	0.001	0.571	NA	NA	0.038	1.000	0.917	1.000
**B*58:01**	0.005	1.000	1.000	1.000	0.033	0.857	1.000	0.621
**B*58:02**	0.001	1.000	NA	NA	0.047	0.967	0.957	NA
**B*73:01**	0.000	1.000	1.000	NA	0.000	NA	NA	NA

Table S3 (continued)

	European Americans				African Americans			
**Allele**	**FREQ**	**SNP2HLA**	**HIBAG**	**HLA*IMP**	**FREQ**	**SNP2HLA**	**HIBAG**	**HLA*IMP**
**B*78:01**	0.000	NA	NA	NA	0.002	0.000	NA	NA
**B*81:01**	0.000	NA	NA	NA	0.011	1.000	NA	NA
**C*01:02**	0.037	0.982	0.986	0.982	0.009	0.833	NA	0.714
**C*02:02**	0.040	0.962	0.970	0.954	0.082	0.127	0.385	0.125
**C*02:06**	0.000	NA	NA	NA	0.001	0.000	NA	NA
**C*02:10**	0.000	NA	1.000	NA	0.000	NA	1.000	NA
**C*03:02**	0.001	0.875	0.833	NA	0.019	0.923	1.000	NA
**C*03:03**	0.058	0.994	0.969	0.985	0.021	0.929	NA	0.471
**C*03:04**	0.089	0.987	0.986	0.970	0.049	0.970	0.933	0.500
**C*03:05**	0.000	NA	NA	NA	0.001	0.000	NA	NA
**C*03:06**	0.000	0.000	NA	NA	0.001	0.000	NA	NA
**C*04:01**	0.095	0.989	0.991	0.991	0.198	0.955	0.962	0.953
**C*04:03**	0.000	0.000	NA	NA	0.001	0.000	NA	NA
**C*04:04**	0.000	0.000	NA	NA	0.001	0.000	NA	NA
**C*04:07**	0.000	0.000	NA	NA	0.001	0.000	NA	NA
**C*04:09**	0.000	NA	NA	NA	0.000	NA	NA	0.000
**C*04:10**	0.000	0.000	NA	NA	0.001	0.000	NA	NA
**C*05:01**	0.099	0.976	0.978	0.971	0.024	0.938	1.000	0.600
**C*06:02**	0.097	0.991	0.991	0.991	0.088	0.949	1.000	0.966
**C*07:01**	0.153	0.990	0.991	0.991	0.129	0.977	0.966	0.973
**C*07:02**	0.149	0.998	0.999	0.999	0.063	0.976	1.000	0.953
**C*07:04**	0.015	0.989	1.000	1.000	0.007	0.800	1.000	1.000
**C*07:26**	0.000	0.000	NA	NA	0.003	0.000	NA	NA
**C*08:01**	0.001	0.333	NA	NA	0.006	0.000	NA	NA
**C*08:02**	0.035	0.980	0.985	0.966	0.039	0.808	1.000	0.808
**C*08:04**	0.000	NA	NA	NA	0.000	NA	1.000	NA
**C*12:02**	0.007	0.976	1.000	1.000	0.001	0.000	NA	NA
**C*12:03**	0.036	0.991	1.000	1.000	0.016	0.909	1.000	0.846
**C*12:04**	0.000	0.000	NA	NA	0.001	0.000	NA	NA
**C*14:02**	0.012	0.973	1.000	0.985	0.027	0.667	0.889	0.375
**C*14:03**	0.000	NA	1.000	NA	0.000	NA	NA	NA
**C*15:02**	0.021	0.960	0.967	0.952	0.004	0.667	NA	0.286
**C*15:04**	0.000	0.000	NA	NA	0.003	0.000	NA	NA
**C*15:05**	0.003	0.867	1.000	1.000	0.027	0.944	1.000	1.000
**C*16:01**	0.038	0.969	0.973	0.940	0.072	0.979	1.000	1.000
**C*16:02**	0.003	0.947	1.000	1.000	0.001	0.000	NA	NA

Table S3 (continued)

	European Americans				African Americans			
**Allele**	**FREQ**	**SNP2HLA**	**HIBAG**	**HLA*IMP**	**FREQ**	**SNP2HLA**	**HIBAG**	**HLA*IMP**
**C*16:04**	0.001	0.571	1.000	NA	0.003	0.000	NA	NA
**C*17:01**	0.006	0.972	1.000	1.000	0.058	0.949	1.000	0.974
**C*18:01**	0.000	0.667	NA	NA	0.034	0.913	0.923	NA
**DPB1*01:01**	0.058	0.991	0.991	0.601	0.295	1.000	0.986	0.880
**DPB1*02:01**	0.127	0.940	0.954	0.903	0.118	1.000	0.950	0.540
**DPB1*02:02**	0.007	0.886	1.000	0.000	0.000	NA	NA	NA
**DPB1*03:01**	0.112	0.879	1.000	0.756	0.062	0.950	1.000	0.882
**DPB1*04:01**	0.424	0.981	0.961	0.960	0.127	0.902	1.000	0.822
**DPB1*04:02**	0.113	0.982	0.986	0.944	0.115	0.932	NA	0.882
**DPB1*05:01**	0.022	0.992	1.000	0.520	0.003	1.000	NA	0.167
**DPB1*05:02**	0.000	NA	NA	0.000	0.000	NA	NA	0.000
**DPB1*06:01**	0.019	0.737	0.827	NA	0.003	1.000	NA	NA
**DPB1*06:02**	0.000	NA	NA	0.000	0.000	NA	0.000	NA
**DPB1*09:01**	0.005	0.806	0.793	0.018	0.001	1.000	NA	0.000
**DPB1*10:01**	0.021	0.960	1.000	0.089	0.006	0.500	NA	0.000
**DPB1*10:40**	0.000	NA	0.000	NA	0.000	NA	0.000	NA
**DPB1*11:01**	0.024	0.993	0.992	0.858	0.031	0.800	1.000	0.684
**DPB1*13:01**	0.020	1.000	1.000	0.812	0.051	0.697	1.000	0.600
**DPB1*14:01**	0.011	0.954	0.919	NA	0.005	1.000	NA	NA
**DPB1*15:01**	0.007	1.000	1.000	NA	0.002	1.000	NA	NA
**DPB1*16:01**	0.007	0.976	0.976	NA	0.002	1.000	NA	NA
**DPB1*17:01**	0.014	0.938	1.000	0.237	0.067	0.837	1.000	1.000
**DPB1*18:01**	0.001	1.000	NA	NA	0.076	1.000	1.000	NA
**DPB1*19:01**	0.007	1.000	0.976	NA	0.003	1.000	NA	NA
**DPB1*20:01**	0.001	0.750	1.000	NA	0.000	NA	NA	NA
**DPB1*23:01**	0.001	0.333	NA	0.000	0.000	NA	NA	NA
**DPB1*26:01**	0.000	1.000	NA	NA	0.000	NA	NA	NA
**DPB1*28:01**	0.000	NA	NA	NA	0.002	1.000	NA	NA
**DPB1*33:01**	0.000	NA	NA	NA	0.002	0.000	NA	NA
**DPB1*40:01**	0.000	NA	NA	NA	0.019	0.917	NA	NA
**DPB1*85:01**	0.000	NA	NA	NA	0.011	1.000	NA	NA
**DQB1*02:01**	0.127	0.980	0.997	0.997	0.134	0.518	0.727	0.953
**DQB1*02:02**	0.098	0.993	0.995	0.991	0.080	1.000	0.905	1.000
**DQB1*03:01**	0.188	0.980	0.982	0.972	0.160	0.971	1.000	0.758
**DQB1*03:02**	0.107	0.992	0.994	0.994	0.038	1.000	1.000	0.923
**DQB1*03:03**	0.049	0.990	1.000	0.997	0.016	1.000	NA	1.000
**DQB1*03:04**	0.001	0.667	1.000	NA	0.000	NA	NA	NA

Table S3 (continued):

	European Americans				African Americans			
**Allele**	**FREQ**	**SNP2HLA**	**HIBAG**	**HLA*IMP**	**FREQ**	**SNP2HLA**	**HIBAG**	**HLA*IMP**
**DQB1*03:05**	0.000	0.000	NA	NA	0.000	NA	NA	NA
**DQB1*03:19**	0.000	NA	0.000	NA	0.000	NA	0.000	NA
**DQB1*04:01**	0.001	0.667	NA	NA	0.000	NA	NA	NA
**DQB1*04:02**	0.026	1.000	0.987	0.974	0.072	1.000	1.000	0.978
**DQB1*05:01**	0.122	0.992	0.993	0.992	0.164	1.000	1.000	1.000
**DQB1*05:02**	0.011	0.970	0.984	0.984	0.031	0.950	1.000	0.857
**DQB1*05:03**	0.026	0.987	1.000	1.000	0.017	1.000	NA	1.000
**DQB1*05:04**	0.002	0.909	1.000	NA	0.000	NA	NA	NA
**DQB1*06:01**	0.006	0.972	1.000	0.971	0.000	NA	NA	0.000
**DQB1*06:02**	0.136	0.986	0.981	0.967	0.206	0.954	0.936	0.926
**DQB1*06:03**	0.054	0.972	0.960	0.962	0.031	0.750	1.000	0.600
**DQB1*06:04**	0.037	0.991	1.000	0.391	0.017	0.909	1.000	0.391
**DQB1*06:09**	0.009	0.982	0.982	1.000	0.033	0.905	1.000	0.615
**DRB1*01:01**	0.092	0.975	0.937	0.886	0.019	0.667	0.714	0.727
**DRB1*01:02**	0.009	0.964	1.000	1.000	0.050	1.000	1.000	1.000
**DRB1*01:03**	0.016	0.958	0.984	0.966	0.000	NA	NA	1.000
**DRB1*03:01**	0.124	0.983	0.986	0.979	0.069	0.932	0.920	0.591
**DRB1*03:02**	0.001	0.750	1.000	NA	0.063	1.000	1.000	NA
**DRB1*03:04**	0.000	0.000	NA	NA	0.000	NA	NA	NA
**DRB1*04:01**	0.106	0.951	0.956	0.869	0.014	0.889	NA	0.172
**DRB1*04:02**	0.007	0.825	0.875	0.826	0.002	0.000	NA	NA
**DRB1*04:03**	0.003	0.778	0.818	0.100	0.000	NA	0.000	NA
**DRB1*04:04**	0.044	0.921	0.922	0.833	0.009	1.000	NA	0.875
**DRB1*04:05**	0.007	0.897	1.000	1.000	0.019	0.750	NA	1.000
**DRB1*04:06**	0.000	0.000	0.000	NA	0.000	NA	NA	NA
**DRB1*04:07**	0.013	0.880	0.839	0.980	0.002	0.000	NA	NA
**DRB1*04:08**	0.003	0.850	1.000	NA	0.002	1.000	NA	NA
**DRB1*04:09**	0.000	0.000	NA	NA	0.000	NA	NA	NA
**DRB1*04:10**	0.000	0.000	NA	NA	0.000	NA	NA	NA
**DRB1*04:11**	0.000	0.000	NA	NA	0.000	NA	NA	NA
**DRB1*04:37**	0.000	0.000	NA	NA	0.000	NA	NA	NA
**DRB1*07:01**	0.135	0.996	0.992	0.989	0.101	0.984	1.000	0.806
**DRB1*08:01**	0.023	0.978	0.977	0.881	0.003	1.000	NA	0.200
**DRB1*08:02**	0.002	0.556	NA	NA	0.000	NA	NA	NA
**DRB1*08:03**	0.003	0.813	1.000	0.500	0.002	1.000	NA	0.000
**DRB1*08:04**	0.002	0.600	1.000	1.000	0.057	0.917	1.000	0.870

Table S3 (continued):

	European Americans				African Americans			
**Allele**	**FREQ**	**SNP2HLA**	**HIBAG**	**HLA*IMP**	**FREQ**	**SNP2HLA**	**HIBAG**	**HLA*IMP**
**DRB1*08:06**	0.000	0.000	NA	NA	0.000	NA	NA	NA
**DRB1*08:10**	0.000	NA	1.000	NA	0.000	NA	NA	NA
**DRB1*09:01**	0.013	0.974	1.000	1.000	0.022	1.000	0.778	1.000
**DRB1*10:01**	0.009	0.962	1.000	1.000	0.025	1.000	1.000	1.000
**DRB1*11:01**	0.055	0.860	0.790	0.721	0.093	0.847	1.000	0.211
**DRB1*11:02**	0.002	0.583	0.833	NA	0.046	0.690	0.591	0.000
**DRB1*11:03**	0.008	0.532	0.000	0.600	0.002	1.000	NA	0.000
**DRB1*11:04**	0.017	0.875	0.899	0.865	0.003	0.500	1.000	0.091
**DRB1*11:06**	0.000	0.000	NA	NA	0.000	NA	NA	NA
**DRB1*11:08**	0.000	0.000	NA	NA	0.000	NA	NA	NA
**DRB1*12:01**	0.016	0.979	1.000	0.957	0.052	0.879	1.000	0.632
**DRB1*12:02**	0.000	0.000	NA	NA	0.002	1.000	NA	NA
**DRB1*13:01**	0.053	0.978	0.980	0.942	0.053	1.000	0.944	0.778
**DRB1*13:02**	0.045	0.985	0.992	0.982	0.075	0.875	0.938	0.800
**DRB1*13:03**	0.010	0.967	1.000	1.000	0.025	1.000	1.000	0.833
**DRB1*13:04**	0.000	0.000	NA	NA	0.000	NA	NA	NA
**DRB1*13:05**	0.001	0.333	NA	NA	0.000	NA	NA	NA
**DRB1*14:01**	0.025	0.947	0.966	0.948	0.020	0.923	NA	0.600
**DRB1*14:02**	0.000	0.000	NA	NA	0.000	NA	NA	NA
**DRB1*14:04**	0.001	0.143	1.000	NA	0.000	NA	NA	0.000
**DRB1*14:05**	0.000	0.000	NA	NA	0.000	NA	NA	NA
**DRB1*14:06**	0.001	0.333	NA	NA	0.000	NA	NA	NA
**DRB1*14:07**	0.000	0.000	NA	NA	0.000	NA	NA	NA
**DRB1*14:15**	0.000	0.000	NA	NA	0.000	NA	NA	NA
**DRB1*15:01**	0.133	0.986	0.977	0.976	0.031	0.800	1.000	0.162
**DRB1*15:02**	0.007	0.949	1.000	0.972	0.003	1.000	NA	0.167
**DRB1*15:03**	0.002	0.667	NA	NA	0.123	1.000	0.982	NA
**DRB1*16:01**	0.010	0.898	0.981	0.897	0.000	NA	NA	0.000
**DRB1*16:02**	0.001	0.500	1.000	NA	0.014	1.000	1.000	NA

HLA indicates human leukocyte antigen; NA, not applicable.

1) NA indicates that the imputation program did not impute the allele
2) Concordance rates were generated using OMNI1 and OMNI5 combined SNP-level data and posterior probability>0.50 for each imputation program
